# History of Extensive Disease Small Cell Lung Cancer Treatment: Time to Raise the Bar? A Review of the Literature

**DOI:** 10.3390/cancers13050998

**Published:** 2021-02-27

**Authors:** Chiara Lazzari, Aurora Mirabile, Alessandra Bulotta, Maria Grazia Viganó, Francesca Rita Ogliari, Stefania Ippati, Italo Dell’Oca, Mariacarmela Santarpia, Vincenza Lorusso, Martin Reck, Vanesa Gregorc

**Affiliations:** 1 Department of Oncology, IRCCS San Raffaele Hospital, via Olgettina 60, 20132 Milano, Italy; Lazzari.Chiara@hsr.it (C.L.); mirabile.aurora@hsr.it (A.M.); bulotta.alessandra@hsr.it (A.B.); vigano.mariagrazia@hsr.it (M.G.V.); ogliari.francesca@hsr.it (F.R.O.); ippati.stefania@hsr.it (S.I.); lorusso.vincenza@hsr.it (V.L.); 2 Department of Radiotherapy, IRCSS San Raffaele Hospital, via Olgettina 60, 20132 Milano, Italy; 3 Medical Oncology Unit, Department of Human Pathology of Adult and Evolutive Age “G. Barresi”, University of Messina, 98121 Messina, Italy; msantarpia@unime.it; 4 Airway Research Center North, German Center for Lung Research, Department of Thoracic Oncology, Lung Clinic, 22927 Grosshansdorf, Germany; m.reck@lungenclinic.de

**Keywords:** small cell lung cancer, chemotherapy, Immunotherapy, extensive disease

## Abstract

**Simple Summary:**

Small cell lung cancer (SCLC) remains the most aggressive form of neuroendocrine tumor of the lung, for which treatment options remain limited. The introduction of immune checkpoint inhibitors has modified for the first time the therapeutic strategies in patients with extensive disease after decades. New therapeutic approaches are required. Deeper knowledge of tumor biology is required to gain new insights into this complex disease.

**Abstract:**

Several trials have tried for decades to improve the outcome of extensive disease small cell lung cancer (ED-SCLC) through attempts to modify the standard treatments. Nevertheless, platinum/etoposide combination and topotecan have remained respectively the first and the second line standard treatments for the last 40 years. With the advent of immunotherapy, this scenario has finally changed. Our review aims to provide an overview of the primary studies on the actual therapeutic strategies available for ED-SCLC patients, and to highlight emerging evidence supporting the use of immunotherapy in SCLC patients.

## 1. Introduction

Small cell lung cancer (SCLC) is an aggressive tumor, with a high mitotic rate and early metastasis occurrence. It is observed in approximately 15% of new cases of lung cancer. Smoking represents the main risk factor for its development. For a long time, SCLC has been classified according to the Veteran’s Administration Lung Cancer Study Group as limited stage (tumor located in the thorax and included in a single radiation field) or extensive stage (when not confined into a single radiation field, or in the presence of distant metastases). To better define patients’ prognosis, in 2009, the International Association for the Study of Lung Cancer proposed the use of the tumor, node, and metastasis (TNM) staging system [1]. Following the introduction of the eighth TNM edition for the classification of non-small cell lung cancer (NSCLC), and the survival analysis of patients with SCLC, classified according to the seventh or the eighth TNM editions, the eighth TNM classification was adopted, and it is currently used [2]. Approximately 60–70% of patients are diagnosed with metastatic disease at the onset [3]. During the last four decades, platinum–etoposide has been the only recognized treatment in the first-line setting. Despite the response rate of 60–80%, responses are not durable, and patients develop resistance and unfortunately die within ten months. Less than 7% of patients are still alive at five years [4,5]. While aiming to improve patients’ outcomes, several treatment strategies have been tested, but with poor results. At odds with NSCLC, where the deep understanding of tumor biology and the identification of actionable molecular alterations have been translated into efficient molecularly targeted therapies, no driver-targetable molecular alterations have been identified in SCLC, and its therapeutic portfolio has not been improved for several years. Recently, immune checkpoint inhibitors have significantly prolonged patient survival, thus resulting in a practice-changing strategy for the first time. 

The current review provides an overview of the progress made in treating patients with extensive disease SCLC (ED-SCLC).

## 2. First Line Chemotherapy in ED-SCLC

Originally, cyclophosphamide, doxorubicin, and vincristine (CAV) represented the standard treatment used in untreated patients with ED-SCLC. For a long time, research has focused on identifying the most effective combinatorial chemotherapy regimen to prolong patients’ survival with acceptable toxicity and good quality of life. Table 1 summarizes the main phase III trials conducted in naive patients with ED-SCLC. 

Because of the high proliferative rate of SCLC, the high chemosensitivity, and the high percentage of relapses, in order to increase tumor-cell death, among the strategies evaluated, the use of alternating non-cross-resistant regimens was tested. A phase III study was designed to compare in 437 patients with ED-SCLC, stratified according to performance status (PS), cisplatin–etoposide (EP) with CAV for four cycles, or the alternation of CAV/EP for six cycles [6]. Overall survival (OS) was the primary end-point of the trial. No significant difference was observed in terms of objective response rate (ORR) or OS. The alternating regimen was associated with a significantly prolonged time to progression (TTP) in comparison to CAV, but with more hematologic toxicity. Moreover, CAV and EP resulted in comparable efficacies in terms of OS, TTP, and ORR. 

Results from a meta-analysis, including data from 36 randomized phase II and III trials, demonstrated, for the first time, OS improvement with etoposide alone, or in combination with cisplatin [7], in patients with ED-SCLC. A further meta-analysis evaluated the differences between cisplatin and carboplatin [8] in terms of efficacy and toxicity. The results were comparable for OS and PFS, and were associated with a different spectrum of toxicity. The included a higher percentage of hematologically adverse events with carboplatin, and more non-hematological events with cisplatin, including nausea, vomiting, and renal failure. Based on these findings and the good efficacy/toxicity ratio of EP, this regimen has become the standard first-line treatment. The choice between cisplatin or carboplatin is secondary to the expected toxicity profile, the organ function, the PS, and the patients’ co-morbidities [8]. 

Different phase III studies have explored the efficacy of adding one or two drugs to EP chemotherapy, the use of alternative compounds to etoposide, such as irinotecan, topotecan or amrubicine, or the use of a high dose of chemotherapy [9]. A phase III trial evaluated the benefit of ifosfamide combined with EP (VIP) in 171 patients with ED-SCLC [10]. Patients were stratified according to PS and randomized between EP or VIP. Patients with brain metastases were eligible and received concurrent whole-brain radiotherapy. The study was designed to demonstrate an OS increase of 50% in the VIP arm compared with the EP regimen. No significant difference was shown in terms of ORR, while prolonged median progression-free survival (PFS) and OS were observed in patients receiving VIP over EP. However, the benefit was less than expected, and VIP was associated with more hematologic and non-hematologic toxicity. Another phase III study tested the advantage of combining cyclophosphamide and epidoxorubicin with EP (PCDE) as a strategy to intensify and improve the therapeutic options in patients with ED-SCLC [11]. Two hundred twenty-six patients lacking previous treatment were randomized between EP or PCDE. Brain metastases represented an exclusion criterion to enter the study. The primary end-point was OS, and the trial was designed to demonstrate a 15% improvement at 1 year with PCDE over EP. At the end of chemotherapy, prophylactic cranial irradiation was recommended for patients with complete responses, while thoracic radiotherapy was suggested for those with partial responses in the case of a residual tumor. PDCE significantly increased ORR, and prolonged PFS and 1-year survival, over EP, with higher hematologic toxicity, and without affecting the quality of life. However, these results did not translate into a practice-changing strategy. In addition, the attempt to use a high dose of EP failed to demonstrate any significant advantage over standard EP in terms of ORR or OS, and was associated with increased toxicity [12]. Another strategy tested was the comparison of a combination therapy containing carboplatin, etoposide, and vincristine with paclitaxel, etoposide and carboplatin, in a phase III study enrolling 614 naive patients with SCLC of any stage [13]. The primary end-point was OS. The results showed a statistically significant increase in median and long-term survival, PFS, and a decrease in toxicities for patients with SCLC receiving a paclitaxel-containing regimen compared with standard chemotherapy.

The preliminary efficacy observed with the topoisomerase I inhibitor irinotecan [14], and the positive findings from a phase II study showing an ORR of 86%, with a median OS of 13.2 months in patients with ED-SCLC receiving cisplatin combined with irinotecan (IC) [15], led to the design of four phase III trials to compare the efficacy of IC to that of EP. All the studies included OS as the primary end-point. The Japanese JCOG 9511, designed to enroll 230 patients, was prematurely closed, following the inclusion of 154 patients [16]. The preliminary results, observed during the interim analysis and showing an OS difference in favor of IC combination, which was further confirmed at the second interim analysis, led to the early termination of patient accrual. The definitive results showed a significant improvement of ORR, PFS, and OS in the IC arm. Patients in the EP arm experienced higher grade 3–4 neutropenia and thrombocytopenia, but lower grade 3–4 diarrhea, than the IC group. However, these results were not confirmed by another phase III trial, designed to compare EP with IC in a larger number of patients from North America [17]. Three hundred thirty-one patients were enrolled. Conversely, from the Japanese trial, no significant difference in terms of ORR, PFS, or OS was observed between the two arms. Different reasons might explain the divergent findings of the two studies, including the differences in the characteristics of the patients enrolled (with a lower percentage of cases with more advanced disease in the JCOG study), the different dose and schedule of IC used, with an increased dose of irinotecan administered in the Japanese trial, and pharmacogenomic differences between the North American and Japanese populations. Similar findings were reported by the phase III SWOG S0124 study, enrolling 651 patients and designed using the same eligibility criteria and treatment regimens as the JCOG 9511 [18]. No difference was observed in ORR, PFS, or OS between the two arms, with less hematologic and greater gastrointestinal toxicity for IC over EP. The different results of the JCOG 9511 trial might be related to the smaller number of patients enrolled, and imbalances in the distribution of patients, including less patients with PS2, more women, and fewer cases with brain metastases in the Japanese study. Moreover, the differences in the ethnicities and the genetic backgrounds of the populations included might have influenced the final results. Conversely, when irinotecan in combination with carboplatin was compared with oral etoposide in combination with carboplatin in a phase III study, enrolling 220 patients with ED-SCLC, irinotecan prolonged OS without affecting the quality of life [19]. More recently, a meta-analysis, including data from six randomized trials [20] enrolling 1476 naive patients with ED-SCLC, compared the efficacy and toxicity of irinotecan/platinum with etoposide/platinum. Two trials were phase II studies, and in two trials carboplatin was used instead of cisplatin. Although the results showed that the combination of irinotecan/platinum improves ORR and prolongs OS over EP, the subgroup meta-analysis performed after excluding the two trials using carboplatin failed to confirm this advantage. The heterogeneity of the population, the different drugs, and the doses used has not allowed for a conclusion on the most efficient regimen, and EP has remained the reference standard treatment for western countries. Another topoisomerase I inhibitor, topotecan, has been explored in combination with cisplatin (TP) in untreated patients with ED-SCLC. Seven hundred and ninety-five patients were enrolled in a phase III trial, designed to assess OS superiority or at least the non-inferiority of TP over EP [21]. Despite a higher ORR, a prolonged PFS, and a comparable OS observed in the TP arm, the higher percentage of hematological toxicities, including anemia and thrombocitopenia requiring transfusions, associated with a higher rate of treatment-related deaths, has not allowed the replacement of standard EP with TP. Similarly, the topoisomerase II inhibitor amrubicin, an anthracycline with the advantage of not determining cardiovascular toxicity, was tested in a phase III study, designed to evaluate its non-inferiority in terms of OS, and eventually its superiority over EP when combined with cisplatin in 300 naive Chinese patients with ED-SCLC [22]. The results showed significantly higher ORR in the AP arm, with comparable PFS and OS, but this was associated with a higher percentage of hematopoietic toxicities in the amrubicine group. 

In conclusion, due to the toxicity and the lack of substantial efficacy, all the alternative regimens tested in patients with untreated ED-SCLC were unsuitable for becoming a new standard, and EP has remained the first therapeutic choice.

## 3. Immune Checkpoint Inhibitors in the Treatment of Patients with ED-SCLC

Recently, in patients with ED-SCLC, the treatment landscape has evolved because of the advent of immune checkpoint inhibitors. The genomic instability of SCLC [23] favors the accumulation of DNA damages, which in turn favor the development of immunogenic clones recognized by the antigen-presenting cells and the dendritic cells. As a consequence, CD8+ T cells are simulated. The activation of the immune system contributes to the killing of tumor cells.

Due to the correlation between SCLC and smoking, many somatic mutations occur. A higher T cells ratio was found in long-term survivors with SCLC compared to patients with recurrent disease [24], thus suggesting the potential efficacy of immune checkpoint inhibitors in SCLC. As such, immunotherapy has been tested in patients with ED-SCLC as a single agent, combined with chemotherapy or different immune checkpoint inhibitors, in the first-line setting, the maintenance, and the second line (Table 2). 

Few data are available regarding the immune composition of SCLC tumors. Retrospective analyses indicate the expression of PD-L1 in a variable percentage of cases (0–71.6%). PD-L1 upregulation has been found in patients with high levels of CD3ε and CD68 mRNA, and in those with increased levels of CD8. CD8 T cells are detectable in 13% of patients [25]. However, a lower percentage of CD8 has been observed in SCLC compared with patients affected by NSCLC. These differences might partly explain the different outcomes obtained with immune checkpoint inhibitors in NSCLC in comparison with SCLC.

Recently, chemotherapeutic agents’ immunologic effects on the tumor microenvironment’s modulation have been established [26]. Preclinical evidence suggests that chemotherapy upregulates PD-L1 on tumor cells, increases the expression of nuclear factor kappa-light on B cells, favors the activation of antigen-presenting cells, and inhibits the infiltration of immunosuppressive cells in the tumor [27,28,29,30]. Based on these findings, clinical trials have been designed to evaluate platinum–etoposide’s synergistic effect with immune checkpoint inhibitors in SCLC [31].

### 3.1. Immune Checkpoint Inhibitors in First Line Setting-CTLA4 Blockade

The anti-CTLA-4 monoclonal antibody ipilimumab was the first immune checkpoint inhibitor evaluated in combination with chemotherapy in untreated patients with ED-SCLC in a phase II study [32]. To define the best timing for chemotherapy and immunotherapy administration, two regimens were tested in 130 patients: ipilimumab concurrent with paclitaxel/carboplatin, or ipilimumab in combination with paclitaxel/carboplatin following two cycles of chemotherapy alone (phased regimen). The primary end-point of the study was immune-related progression-free survival (irPFS). Results showed improved irPFS in the group receiving the phased regimen over chemotherapy alone, while no difference was observed with the concurrent regimen, thus suggesting that induction chemotherapy might favor the release of tumor antigens, thus resulting in a more effective treatment strategy. Based on these promising findings, the phase III CA 184-156 study was designed to compare ipilimumab or placebo in combination with etoposide and platinum [33]. The phased regimen was selected. One thousand one hundred and thirty-two patients were enrolled to demonstrate an OS improvement in the experimental arm. Conversely, from what was expected, ipilimumab did not result in a statistically significantly improved OS over chemotherapy alone, and immunotherapy did not enter into the therapeutic armamentarium of patients with ED-SCLC.

### 3.2. Immune Checkpoint Inhibitors in First-Line Setting-PD-1/PD-L1 Blockade

Previous reports showed that PD-L1 is expressed in immune cells infiltrating the SCLC stroma, thus suggesting the potential of enhancing tumor-specific T-cell immunity by targeting the PD-1/PD-L1 pathway. Three phase III studies and one phase II study have been designed to evaluate the safety and efficacy of atezolizumab, durvalumab, pembrolizumab and nivolumab in combination with platinum–etoposide-based chemotherapy. 

The efficacy of atezolizumab combined with carboplatin etoposide was evaluated in the phase III IMpower 133, designed to demonstrate the OS and PFS improvement of the combination over carboplatin–etoposide in 403 patients with naïve ED-SCLC. Patients were stratified according to sex, ECOG PS, and the presence of brain metastases [34]. In the presence of clinical benefit, patients were allowed to continue therapy beyond progression. Prophylactic cranial irradiation was allowed following the first four cycles, during the atezolizumab/placebo maintenance phase. The trial reached the primary end-points, showing a statistically significant reduction in the risk of death of 30%, and a progression of 23% in patients receiving atezolizumab. One-year OS rate was 13% higher in the atezolizumab group than in the placebo group (51.7 vs. 38.2%). The benefit was independent of the biomarkers analyzed, since neither PD-L1 expression nor blood tumor molecular burden were predictive of the outcome, and this was observed in all the subgroups analyzed except for those patients with treated brain metastases. However, this was an exploratory, and not a pre-planned, analysis performed on a limited number of cases, and no definitive conclusion can be drawn. Based on these results, the combination of carboplatin, etoposide and atezolizumab has become the new referral standard treatment for naive patients with ED-SCLC. 

The CASPIAN trial was a randomized, phase III study, designed to compare the efficacy of the PD-L1 inhibitor durvalumab, with or without the CTLA-4 inhibitor tremelimumab, in combination with platinum–etoposide in 805 patients with untreated ED-SCLC [35]. In the case of progression, continuation of treatment was allowed in the presence of clinical benefits. The co-primary end-points were OS for durvalumab platinum–etoposide versus chemotherapy, and for durvalumab tremelimumab platinum–etoposide versus chemotherapy. The adding of durvalumab tremelimumab did not significantly prolong OS over chemotherapy. Conversely, as already observed in the IMpower 133 trial, the combination of durvalumab with platinum–etoposide significantly improved OS. No significant difference was evidenced in terms of PFS between the treatment arms. A survival benefit was also found with durvalumab in the group of patients with untreated brain metastases. These results confirm the lack of any additional benefit with the CTLA-4 blockade in unselected patients with ED-SCLC. Based on these findings, durvalumab in combination with cisplatin or carboplatin etoposide has been approved by the Food and Drug Administration (FDA) and the European Medicine Agency (EMA) for use in untreated patients with ED-SCLC.

The phase III KEYNOTE 604 trial was designed to investigate the efficacy of the PD-1 inhibitor pembrolizumab, combined with platinum–etoposide, over chemotherapy alone in 453 naive patients with ED-SCLC [36]. The co-primary end-points were OS and PFS. The trial reached one of its primary end-points, showing a significant PFS improvement in chemotherapy and immunotherapy, which did not translate into prolonged OS, despite the numerical benefit in OS being comparable to those achieved by IMpower 133 and CASPIAN. However, double the percentage of patients in the experimental arm were alive at two years compared with chemotherapy alone, thus suggesting that a subgroup of patients obtain a durable benefit from the combination. Compared to the IMpower 133 and the CASPIAN trials, more patients with brain metastases (with ECOG PS of 1) with large tumor dimensions, elevated LDH, and more metastatic sites were enrolled in the KEYNOTE 604 trial. These differences in the populations might have influenced the final results.

Finally, the PD-1 inhibitor nivolumab was tested in combination with platinum–etoposide in the phase II EA5161 trial, enrolling 160 naive patients with ED-SCLC [37]. The study reached its primary end-point, showing a significant PFS improvement in the experimental arm. 

In conclusion, the durable benefit observed with the addition of PD-1 or PD-L1 inhibitors to platinum–etoposide has modified the therapeutic strategies recommended in patients with ED-SCLC after several decades. This is particularly remarkable in such an aggressive disease, where, historically, it has been challenging to overcome the long-term survival benefit obtained with platinum–etoposide. 

### 3.3. Immune Checkpoint Inhibitors as a Maintenance Strategy

Aiming to prolong survival, immune checkpoint inhibitors have been tested in the maintenance setting in those patients with stable or responsive ED-SCLC following four to six cycles of platinum–etoposide, but with poor results.

Pembrolizumab was evaluated in a phase II study, designed to demonstrate in 45 patients a PFS improvement of 50%, compared with historical data [38]. Pembrolizumab was administered within eight weeks from the end of chemotherapy. Despite no significant PFS or OS improvement being observed among the patients enrolled, the 1-year PFS and OS rates were 13% and 37%, respectively, thus suggesting that a subgroup of patients might receive durable benefits from this strategy.

Similar results were found in the phase III CA 451 study, designed to compare nivolumab with the combination of ipilimumab + nivolumab or a placebo in 834 patients with ED-SCLC, who did not progress following four cycles of platinum-based chemotherapy [39]. The study failed to demonstrate OS improvement in nivolumab + ipilimumab patients over placebo patients, and of nivolumab over placebo.

These findings suggest that the timing of checkpoint inhibitors administration is important in improving the survival benefit in patients with ED-SCLC, and a maintenance strategy with immune checkpoint inhibitors is not suggested.

### 3.4. Immune Checkpoint Inhibitors in Recurrent Patients

Controversial results have been observed in the studies evaluating the efficacy of pembrolizumab, nivolumab, durvalumab and atezolizumab in patients progressing after platinum–etoposide-based chemotherapy.

The activity of pembrolizumab as a monotherapy in the second/third-line setting was assessed in two trials, the phase Ib KEYNOTE 028 trial and the phase II KEYNOTE 058 trial. The KEYNOTE 028 trial [40] was a multi-cohort phase Ib study, designed to define the preliminary effects of pembrolizumab in different cohorts of patients with solid tumors. The presence of PD-L1 expression in at least 1% of tumors and inflammatory cells represented one of the inclusion criteria to enter the study, the primary end-point of which was to determine the percentage of objective responses. One of the cohorts included 24 patients with ED-SCLC progressing to at least one previous line of chemotherapy. Among these, approximately half of them had received the second line with topotecan or irinotecan. An ORR of 33.3% was identified, with a median duration of response of 19.4 months, thus suggesting a promising clinical activity of pembrolizumab in this setting. In order to confirm these findings in a larger population, the phase II KEYNOTE 058 trial enrolled patients progressing to standard treatment [41]. Similarly, to the KEYNOTE 028 trial, the primary end-point here was the percentage of objective response. Differently from KEYNOTE 028, here, PD-L1 positivity was not an inclusion criterion, even though to enter the trial the collection of evaluable tumor samples was mandatory, in order to centrally test PD-L1 expression. One hundred and seven patients with ED-SCLC were included, and an ORR of 18.7% was registered. The percentage of responses was six times higher in PD-L1-positive compared to PD-L1-negative tumors (35.7% vs. 6.0%), and longer OS was observed in PD-L1-positive patients (14.6 vs. 7.7 months). A pooled analysis of the two studies, including only those patients who received two lines of chemotherapy (83/131), showed that 88% of responder patients had PD-L1-positive tumors, thus suggesting the importance of selecting patients according to PD-L1 expression [42].

Nivolumab was tested in the CheckMate 032 and CheckMate 331 trials. 

The CheckMate 032 was a phase I/II study investigating the activity and safety of nivolumab as a monotherapy, or in combination with ipilimumab, in metastatic patients with different solid tumor types [43]. Two hundred and sixteen patients with LD or ED-SCLC progressing after one or more previous regimens were enrolled into four cohorts in a sequential manner. In each cohort, a different regimen was administered, including nivolumab 1 mg/kg + ipilimumab 1 mg/kg, nivolumab 1 mg/kg + ipilimumab 3 mg/kg, or nivolumab 3 mg/kg + ipilimumab 1 mg/kg. The combination was continued for four cycles, followed by nivolumab until progression or unacceptable toxicity. The percentage of objective response was the primary end-point of the trial. The ORR was 10% in patients receiving nivolumab, 23% in those included in the nivolumab 1 mg/kg + ipilimumab 3 mg/kg cohort, 19% in those receiving nivolumab 3 mg/kg + ipilimumab 1 mg/kg, and 33% in the group treated with nivolumab 1 mg/kg + ipilimumab 1 mg/kg. Tissue was available for PD-L1 assessment in 69% of cases. Conversely from what was observed with pembrolizumab, tumor responses occurred independently of PD-L1 expression. Despite the limited number of patients enrolled, the preliminary analysis showed a similar efficacy between platinum-sensitive and -resistant patients, and between those previously treated with one, or two or more, lines of therapy [44]. To confirm these findings, the phase III CheckMate 331 trial was designed. However, the study did not reach the primary end-point of showing the improved OS of nivolumab over chemotherapy (topotecan or amrubicin) in 569 patients with SCLC previously treated with one line of platinum–etoposide [45].

The efficacy of durvalumab was evaluated in a phase I/II study, enrolling 21 patients with pretreated ES-SCLC [46], whose primary objective was to determine the safety profile of durvalumab. An ORR of only 9.5% was registered.

Finally, atezolizumab did not demonstrate any advantage over topotecan or re-induction of chemotherapy in 73 patients with pretreated ED-SCLC, enrolled in the phase II IFCT-1603 trial [47]. 

In conclusion, immune checkpoint inhibitors have not shown a significant advantage in relapsing patients with ED-SCLC, despite the fact that a subgroup of them might experience a durable clinical benefit. Biomarkers able to select those patients who benefit more have not been identified yet, despite results from the KEYNOTE 028 and the KEYNOTE 058 studies suggesting that PD-L1 expression could be considered. However, these findings were not confirmed in the studies testing nivolumab, where the activity was observed irrespective of PD-L1 status. Finally, in the trials evaluating durvalumab and atezolizumab, patients were not selected according to PD-L1 expression, and no definitive conclusions could be drawn [48].

## 4. Second Line and Beyond 

Those patients relapsing or progressing during first-line treatment are classified as refractory. In the case of progression within 3 months, they are considered platinum-resistant. If relapse is observed after 3 months (the last cycle of first-line chemotherapy), they are considered platinum-sensitive.

According to ESMO guidelines, in sensitive patients, a rechallenge with drugs used in first-line therapy is allowed. However, the lack of PFS and OS benefit was observed in a retrospective analysis, including data from 65 patients with sensitive ED-SCLC [49]. On the other hand, a systematic analysis, including data from 1692 sensitive and refractory patients with SCLC, showed higher ORR and longer OS in sensitive patients [50]. 

Different compounds have been tested as therapeutic strategies in refractory patients with ED-SCLC (Table 3).

Among these, topotecan failed to demonstrate improved efficacy in terms of ORR over CAV in a randomized trial enrolling 211 relapsing patients with ED-SCLC. No significant difference was observed in terms of ORR, PFS or OS between the two arms, although a greater proportion of patients in the topotecan group showed improved symptoms [51]. Thanks to these findings, topotecan was approved for the second-line treatment of sensitive SCLC patients. 

The efficacy of topotecan was compared to amrubicin in a phase II trial enrolling 76 patients with ED-SCLC who were sensitive to previous first-line platinum-based chemotherapy [52]. ORR was the primary end-point of the study. The results showed significantly higher ORR with amrubicin, and longer, but non-statistically significant, OS. The limited number of patients enrolled in the phase II study and the lack of independent confirmation of responses might have influenced the final results. A phase III trial is currently ongoing to confirm (or not) these data. 

The promising results from a phase II study with lurbinectedin in 105 refractory patients with SCLC, showing an ORR of 35% with a median response duration of 5.3 months [54]. secured lurbinectedin the accelerated approval of the FDA for patients with metastatic SCLC previously treated with platinum-based chemotherapy. However, the phase III ATLANTIS trial, comparing lurbinectedin in combination with doxorubicin versus the physician’s choice of topotecan or CAV, failed to demonstrate an OS improvement in 613 patients with SCLC progressing to one previous line of platinum-based chemotherapy.

Alternative strategies to chemotherapy and immune check point inhibitors, including compounds targeting angiogenesis, have been tested. Among these, a single-arm phase II trial safely combined NGR-hTNF, a vascular-targeting agent (0.8 μg/m^2^), which increases intra-tumoral chemotherapy penetration and T-lymphocyte infiltration [55], with doxorubicin (75 mg/m^2^ every 3 weeks), showing manageable toxicity and promising activity in unselected platinum-resistant or platinum-sensitive patients with relapsed ED-SCLC. The primary end-point was PFS. Safety, ORR and OS were the secondary end-points. The median PFS was longer in platinum-sensitive compared to platinum-resistant patients (4.1 vs. 2.7 months, respectively). Prolonged OS was observed in those patients with increased lymphocyte counts [53]. The preclinical data indicate that NGR-hTNF modifies the composition of the tumor microenvironment, including the infiltration of CD8+ T cells, and favors the secretion of cytokines and chemokines [55], thus suggesting that NGR-hTNF might enhance the efficacy of immune checkpoint inhibitors [56]. NGR-hTNF combined with immunotherapy might represent a strategy to be evaluated in patients with relapsed SCLC. 

All the agents tested have shown modest activity. To date, topotecan represents the only approved drug for the second-line treatment of patients with ED-SCLC. 

## 5. Discussion and Future Directions

**Figure 1:**
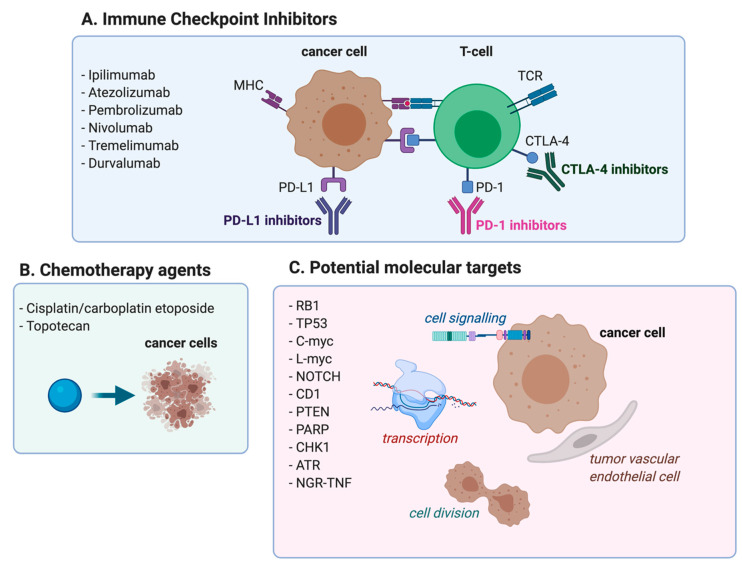
(**A**) Immunotherapy treatment strategies currently approved or tested in patients with ED-SCLC. (**B**) Approved chemotherapy treatments for patients with ED-SCLC. (**C**) Potentially targetable and identified molecular pathways in ED-SCLC.

SCLC remains the most aggressive form of neuroendocrine tumor of the lung, for which treatment options remain limited. The characterization of the tumor biology of SCLC has been challenging, mainly due to the limited availability of tumor tissue, since surgery represents an option confined to a small number of cases. The high number of somatic mutations, generally including loss of function mutations or deletions in tumor suppressor genes, have further increased the difficulty of developing selected targeted therapies. Alterations in the genes involved in cell cycle regulation or DNA damage response, including RB transcriptional corepressor 1 (RB1) and tumor protein p53 (TP53), are commonly observed in SCLC [57]. The amplification of the chromosomal regions, including L-myc [58] or C-myc [59], the overexpression of the cyclin D1 [57], the alteration of PTEN [60] or of genes involved in transcriptional regulation and chromatin modification, the presence of inactivating mutations in NOTCH, and the overexpression of genes responsible for DNA damage response, represent some of the molecular aberrations characterizing the genomic landscape of SCLC [61]. To date, the majority of the trials investigating a molecular approach have provided disappointing results, mainly because the patients enrolled were not selected according to specific driver molecular alterations. The introduction of immune checkpoint inhibitors has improved systemic treatments after decades (Figure 1). However, patients’ prognoses remain dismal, and new therapeutic approaches are required. Tissue collection is strongly advocated to gain new insights into the biology of this complex disease. The implementation of non-invasive methods, including the analysis of circulating tumor DNA and the use of circulating tumor cells (CTC), might become tools to overcome the inadequate amounts of tumor samples, in order to dissect the pathogenesis of SCLC and discover new targetable molecular alterations. Preclinical studies using CTC-derived explants (CDX) might be useful to identify the bypass signaling of acquired resistance, and design combinatorial strategies to overcome these pathways.

## Figures and Tables

**Table 1:** Phase III trials exploring chemotherapy in first-line setting of patients wing extensive disease small cell lung cancer (ED-SCLC).

Author (Ref)	Treatment	Primary End-Point	OS (m)	*p*	TTP/PFS (m)	*p*	ORR (%)
Ihde [12]	high EP standard EP	RR	10.7 11.4	0.68	7.0 6.9	0.96	86 83
Roth [6]	EP CAV EP/CAV	OS	4.3 4.0 5.2	0.425	4.3 4.0 5.2	0.052	61 51 60
Loehrer [10]	EP VIP	OS	7.3 9.1	0.045	6.0 6.8	0.039	67 73
Pujol [11]	EP PCDE	OS	9.3 10.5	0.0067	7.2 6.3	<0.00001	61 76
Reck [13]	TEC CEV	OS	12.7 11.7	0.024	8.1 7.5	0.033	72.1 69.4
Noda [16]	IC EP	OS	12.8 9.4	0.002	6.9 4.8	0.03	84.4 67.5
Hanna [17]	IC EP	OS	10.2 9.3	0.68	4.1 4.6	0.37	48.7 43.6
Lara [18]	IC EP	OS	9.1 9.9	0.071	5.8 5.2	0.07	60 57
Hermes [19]	CBDCA + E ^(^*^)^ CBDCA + I	OS	8.5 7.1	0.02	-	-	-
Fink [21]	TP EP	OS	44.9 weeks 40.9 weeks	0.029	27.4 weeks 24.3 weeks	0.01	55.5 45.5
Sun [22]	AP EP	OS	11.8 10.3	0.008	6.8 5.7	0.035	69.8 57.3

EP: cisplatin–etoposide. CAV: cyclophosphamide, doxorubicin and vincristine. VIP: ifosfamide, cisplatin, etoposide. PDCE: cyclophosphamide, epidoxorubicin, cisplatin, etoposide. CEV: carboplatin, etoposide, and vincristine. TEC: paclitaxel, etoposide, and carboplatin. IC: irinotecan, cisplatin. CBDCA: carboplatin. E: etoposide. I: irinotecan. * oral. TP: topotecan, cisplatin. AP: amrubicin, cisplatin. OS: overall survival. PFS: progression-free survival. TTP: time to progression. m: months.

**Table 2:** Clinical trials exploring immune checkpoint inhibitors in patients with ED-SCLC.

Author	Treatment	Setting	Primary End-Point	OS (m)	*p*	PFS (m)	*p*
Reck [32]	Phased I + PC Concurrent I + PC PC	I line	irPFS	12.5 9.1 10.5	0.13 0.41	6.4 5.6 5.2	0.03 0.11
Reck [33]	Ipilimumab + PE PE	I line	OS	11 10.9	0.37	4.6 4.4	-
Horn [34]	Atezolizumab + CE CE	I line	OS PFS	12.3 10.3	0.007	5.2 4.3	0.02
Goldman [35]	DPE PE DTPE	I line	OS	12.9 10.5 10.4	0.0032 0.0045	5.1 5.4 4.9	-
Rudin [36]	Pembrolizumab + PE PE	I line	OS PFS	10.8 9.7	0.0164	4.5 4.3	0.0023
Leal [37]	Nivolumab + PE PE	I line	PFS	11.3 9.3	0.14	5.5 4.6	0.012
Gadgeel [38]	Pembrolizumab	maintenance	PFS	9.6	-	1.4	-
Owonikoko [39]	Nivolumab Nivolumab + Ipilimumab	maintenance	OS	10.4 9.2	-	1.9 1.7	-
Ott [40]	Pembrolizumab	II/III line	ORR	9.7	-	1.9	-
Chung [41]	Pembrolizumab	II/III line	ORR	9.1	-	2.0	-
Antonia [43]	Nivolumab Nivolumab 1 mg/kg + Ipilimumab 1 mg/kg Nivolumab 1 mg/kg + Ipilimumab 3 mg/kg Nivolumab 3 mg/kg + Ipilimumab 1 mg/kg	II/III line	ORR	5.7 4.7 (#)	-	1.4 1.5 (#)	-
Reck [45]	Nivolumab Topotecan/amrubicine	II line	OS	7.5 8.4	0.11	1.4 3.8	-
Goldman [46]	Durvalumab	I/II line	safety	4.8	-	1.5	-
Pujol [47]	Atezolizumab Topotecan/PE	II line	ORR at 6 months	9.5 8.7	0.60	1.4 4.3	-

I: ipilimumab. PC: paclitaxel, carboplatin. CE: etoposide, carboplatin. DPE: durvalumab, platinum*, etoposide. DTPE: durvalumab, tremilimumab, platinum*, etoposide. * cisplatin or carboplatin was allowed according to investigators’ choice. PFS: progression-free survival. ORR: objective response rate. (#): includes all the patients receiving nivolumab + ipilimumab irrespective of the schedule used.

**Table 3:** Clinical trials exploring second-line therapies in patients with ED-SCLC.

Author	Regimen	Primary End-Point	OS (m)	ORR %	PFS (m)
von Pawel [51]	Topotecan CAV	PFS	25.0 24.7	24.3 18.3	3.3 3.0.
Jotte [52]	Topotecan Amrubicin	ORR	7.8 7.5	15 44	3.3 4.5
Gregorc [53]	NGR-hTNF + Doxorubicin	PFS	13.1	25	3.2

PFS: progression-free survival. ORR: objective response rate.

## Data Availability

N/A.
